# Impact of Heparanase and the Tumor Microenvironment on Cancer Metastasis and Angiogenesis: Basic Aspects and Clinical Applications

**DOI:** 10.5041/RMMJ.10019

**Published:** 2011-01-31

**Authors:** Israel Vlodavsky, Michael Elkin, Neta Ilan

**Affiliations:** 1 Cancer and Vascular Biology Research Center, Rappaport Faculty of Medicine, Technion, Haifa 31096, Israel; and; 2 Sharett Institute, Hadassah-Hebrew University Medical Center, Jerusalem 91120, Israel

**Keywords:** Heparanase, heparan sulfate, extracellular matrix, angiogenesis, metastasis, inflammation, myeloma, signaling, C-domain, matrix metalloproteinase

## Abstract

Heparanase is an endo-β-D-glucuronidase that cleaves heparan sulfate (HS) side chains at a limited number of sites, activity that is strongly implicated with cell invasion associated with cancer metastasis, a consequence of structural modification that loosens the extracellular matrix barrier. Heparanase activity is also implicated in neovascularization, inflammation, and autoimmunity, involving migration of vascular endothelial cells and activated cells of the immune system. The cloning of a single human heparanase cDNA 10 years ago enabled researchers to critically approve the notion that HS cleavage by heparanase is required for structural remodeling of the extracellular matrix (ECM), thereby facilitating cell invasion. Heparanase is preferentially expressed in human tumors and its over-expression in tumor cells confers an invasive phenotype in experimental animals. The enzyme also releases angiogenic factors residing in the tumor microenvironment and thereby induces an angiogenic response *in vivo*. Heparanase up-regulation correlates with increased tumor vascularity and poor postoperative survival of cancer patients. These observations, the anticancerous effect of heparanase gene silencing and of heparanase-inhibiting molecules, as well as the unexpected identification of a single functional heparanase suggest that the enzyme is a promising target for anticancer drug development. Progress in the field expanded the scope of heparanase function and its significance in tumor progression and other pathologies such as inflammatory bowel disease and diabetic nephropathy. Notably, while heparanase inhibitors attenuated tumor progression and metastasis in several experimental systems, other studies revealed that heparanase also functions in an enzymatic activity-independent manner. Thus, point-mutated inactive heparanase was noted to promote phosphorylation of signaling molecules such as Akt and Src, facilitating gene transcription (i.e. VEGF) and phosphorylation of selected Src substrates (i.e. EGF receptor). The concept of enzymatic activity-independent function of heparanase gained substantial support by elucidation of the heparanase C-terminus domain as the molecular determinant behind its signaling capacity and the identification of a human heparanase splice variant (T5) devoid of enzymatic activity, yet endowed with protumorigenic characteristics. Resolving the heparanase crystal structure will accelerate rational design of effective inhibitory molecules and neutralizing antibodies, paving the way for advanced clinical trials in patients with cancer and other diseases involving heparanase.

## PREFACE

The extracellular matrix (ECM) is a heterogeneous mixture of proteins and polysaccharides that surrounds cells, providing physical support for cellular organization into tissue and organs. Traditionally, the ECM was regarded as an inert scaffold providing a structural framework for cells to form tissues and organs. Specifically, our research focuses on heparan sulfate (HS) glycosaminoglycan (GAG), one of the most important subsets of the ECM and cell surface molecules, shown to have a pronounced effect on fundamental biological processes, ranging from development and formation of blood vessels to cell invasion and viral infection. While 4 and 20 building-blocks make nucleic acids and proteins, respectively, 32 disaccharide building-blocks make up these complex, highly acidic, and information-dense biopolymers. The chemical heterogeneity and structural complexity of GAGs make investigations of these molecules most challenging, with fundamental questions arising as to how topological positioning and function of cells and tissues are regulated by GAGs.

Back in 1979, we were among the first to realize that the ECM plays an active role in orchestrating cellular responses to both normal and pathological situations.^1^,^2^ The emerging picture was one of active interplay between cells and ECM where cells synthesize the matrix components which in turn dictate and regulate cell shape and function.^1^,^2^ The ECM network of proteins, glycoproteins, and proteoglycans provides adherent cells with structural support and biochemical cues that regulate cell fate and function. We developed a straightforward approach to coat plastic surfaces with ECM deposited by cultured endothelial cells and demonstrated that this naturally produced ECM closely resembles the subendothelial basement membrane (BM) *in vivo*.^2^,^3^ This ECM and the more commonly used three-dimensional tumor-derived BM-like substrate (Matrigel^™^; BD Biosciences)^4^ are being applied to sustain cell proliferation, differentiation, and survival *in vitro,* retaining the *in-vivo* characteristics.^5^ The ECM/Matrigel system is also widely used to study tumor cell invasion and vascular sprouting.

Tumor cell invasion and spread through the blood and lymphatics (metastasis) is the hall-mark of malignant disease and the greatest impediment to cancer cure. Metastasis is a multistage process that requires cancer cells to escape from the primary tumor, survive in the circulation, seed at distant sites, and grow. Each of these processes involves rate-limiting steps that are influenced by the malignant and non-malignant cells of the tumor microenvironment.^6^,^7^ A tumor must continuously recruit new capillary blood vessels (a process called angiogenesis) to sustain itself and grow.^8^ Moreover, the new blood vessels embedded in the tumor serve as a gateway for tumor cells to enter the circulation and metastasize to distant sites.^7^ Numerous studies have shown that metastasis formation depends on the ability of tumor cells to invade blood vessel walls and tissue barriers in a process involving enzymes capable of digesting ECM components. Attention focused on serine (i.e. plasminogen activators) and cysteine (i.e. cathepsins) proteases as well as matrix metalloproteinases (MMPs).^9^

These enzymes, whose substrates include major components of the ECM, including collagens, laminin, fibronectin, and vitronectin, are often up-regulated in metastatic cancers. It was originally thought that their role was simply to break down tissue barriers, enabling tumor cells to invade through stroma and blood vessel at primary and secondary sites. Subsequent studies revealed that MMPs and plasminogen activators also participate in angiogenesis and are selectively up-regulated in proliferating endothelial cells.^10^ Furthermore, these proteases can contribute to the sustained growth of established tumor foci by cleavage of the ectodomain of membrane-bound proforms of growth factors, releasing peptides that are mitogens for tumor cells and/or vascular endothelial cells.^10^

**Figure 1:**
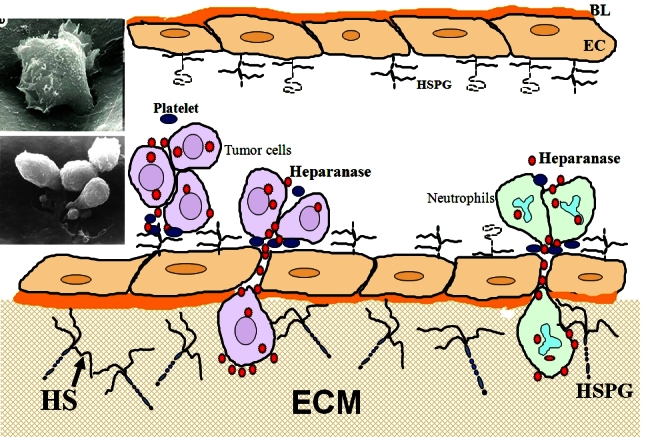
Heparanase-mediated extravasation of blood-borne cells. Heparanase expressed by tumor cells (left) and neutrophils (right) promotes cell invasion in between adjacent vascular endothelial cells (EC) and through their underlying basal lamina (BL) into the extracellular matrix (ECM). Left: Scanning electron micrographs showing invasion of T lymphoma cells, in the absence (top) or presence (bottom) of platelets, through a monolayer of cultured vascular EC. HS, heparan sulfate; HSPG, heparan sulfate proteoglycan.

The other chief components of the ECM are glycosaminoglycan polysaccharides, of which heparan sulfate (HS) is the most abundant in the subepithelial and subendothelial basement membranes. Heparan sulfate proteoglycans (HSPGs) are composed of a protein core covalently linked to heparan sulfate (HS) glycosaminoglycan chains that interact closely with other ECM components.^11^,^12^ These linear saccharide chains are cleaved by an endoglycosidase activity, heparanase, that degrades the HS side chains of HSPGs.^13^–^15^ Normally, the enzyme is found mainly in platelets, mast cells, placental trophoblasts, keratinocytes, and leukocytes. Heparanase released from activated platelets and cells of the immune system facilitates extravasation of inflammatory cells. It also stimulates endothelial mitogenesis, primarily through release of HS-bound growth factors (i.e. fibroblast growth factor (FGF), hepatocyte growth factor (HGF), vascular endothelial growth factor (VEGF)) residing in the ECM.^16^,^17^ Tumor cells appear to use the same molecular machinery during metastasis and neoangiogenesis (Figure 1). Thus, the normal physiological functions of proteases and heparanases in embryonic morphogenesis, wound-healing, tissue repair, and inflammation have been effectively “hijacked” by tumor cells.

Evidence indicates that heparanase not only assists in the break-down of ECM but also is involved in regulating the bioavailability and activity of growth factors and cytokines. Briefly, various heparin-binding growth factors are sequestered by HS in the ECM, providing a localized, readily accessible depot, protected from proteolytic degradation,^18^,^19^ yet available to activate cells after being released by heparanase. It is conceivable that release of tissue-specific growth factors may be involved in the organ selectivity of metastasis. Although these well documented phenomena were investigated by us and other groups, it has taken nearly 15 years to isolate and clone the heparanase gene, mainly because of instability of the enzyme(s) and the difficulty in designing specific, quantitative assays. The cDNA sequences of the first and apparently only mammalian heparanase, isolated from human placenta^14^ and platelets,^15^ have been reported in 1999, and putative precursor and active recombinant enzymes have been expressed. Subsequent studies demonstrated that the heparanase DNA sequences derived from normal and tumor cells (which undoubtedly represent the same gene) are unique. It soon became apparent that cloning and functional characterization of the long sought-after heparanase opens a new chapter in the understanding and potential manipulation of metastasis, angiogenesis, and inflammatory processes. The present review article summarizes our long-term and on-going research on the biology of the heparanase enzyme, emphasizing its clinical relevance.

## HEPARAN SULFATE PROTEOGLYCANS

HSPGs are composed of core protein to which glycosaminoglycan (GAG) side chains are covalently attached. GAGs are linear polysaccharides consisting of a repeating disaccharide generally of an acetylated amino sugar alternating with uronic acid. Units of N-acetylglucosamine and glucuronic/iduronic acid form heparan sulfate (HS).^11^,^12^ The polysaccharide chains are modified at various positions by sulfation, epimerization, and N-acetylation, yielding clusters of sulfated disaccharides separated by low or non-sulfated regions.^12^,^20^ The sulfated saccharide domains provide numerous docking sites for a multitude of protein ligands, ensuring that a wide variety of bioactive molecules (e.g. heparin-binding growth factors, cytokines, chemokines, lipoproteins, enzymes) bind to the cell surface and ECM^11^,^21^ and thereby function in the control of normal and pathological processes, among which are morphogenesis, tissue repair, inflammation, vascularization, and cancer metastasis.^11^,^12^,^22^ Two main types of cell surface HSPG core proteins have been identified: the transmembrane syndecan with four isoforms,^11^ and the glycosylphosphatidyl inositol (GPI)-linked glypican with six isoforms.^23^ Two major types of ECM-bound HSPG are found: agrin, abundant in most basement membranes, primarily in the synaptic region;^24^ and perlecan, with a wide-spread tissue distribution and a very complex modular structure.^20^

From mice to worms, embryos that lack HS die during gastrulation, suggesting a critical developmental role for HSPGs. HSPG function is not limited to developmental processes but plays key roles in numerous biological settings, including cytoskeleton organization and cell–cell and cell–ECM interactions.^22^,^25^ HSPGs exert their multiple functional repertoires via several distinct mechanisms that combine structural, biochemical, and regulatory aspects. By interacting with other macromolecules, such as laminin, fibronectin, and collagens I and IV, HSPGs contribute to the structural integrity, self-assembly, and insolubility of the ECM and basement membrane, thus intimately modulating cell–ECM interactions.^11^,^26^,^27^ Biochemically, HSPGs often facilitate the biological activity of bound ligands by actively participating in receptor–ligand complex formation.^28^ In other cases, HSPGs mediate cellular uptake and catabolism of selected ligands,^28^ and/or sequester polypeptides to the ECM and cell surface, generally as an inactive reservoir.^18^,^29^–^32^ Cleavage of HSPGs would ultimately release these proteins and convert them into bioactive mediators, ensuring rapid tissue response to local or systemic cues.

Accumulating evidence indicates that HSPGs act to inhibit cellular invasion by promoting tight cell–cell and cell–ECM interactions, and by maintaining the structural integrity and self-assembly of the ECM.^33^,^34^ Notably, one of the characteristics of malignant transformation is down-regulation of GAG biosynthesis, especially of the HS chains.^33^,^34^ Low levels of cell surface HS also correlate with high metastatic capacity of many tumors.

## MAMMALIAN HEPARANASE

**Figure 2:**
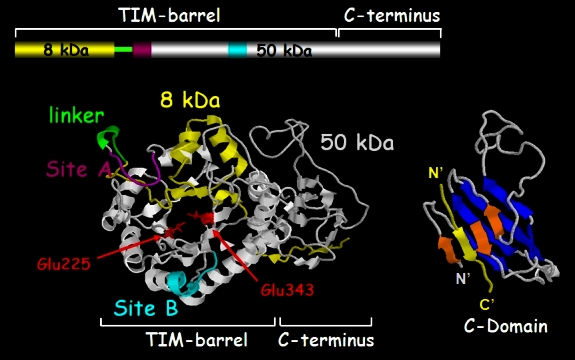
Predicted model of the active heparanase heterodimer showing the 50 + 8 kDa heparanase subunits, TIM-barrel and C-terminus domains, active site (Glu_225_ and Glu_343_, red), and heparin-binding domains (sites A and B). Right: Detailed structure of the C-domain.

Enzymatic activity capable of cleaving glucuronidic linkages and releasing polysaccharide chains resistant to further degradation by the enzyme was first identified by Ogren and Lindahl.^35^ The physiological function of this activity was initially implicated in degradation of macromolecular heparin to physiologically active fragments.^35^,^36^ Heparanase is an endo-β-glucuronidase that cleaves HS side chains presumably at sites of low sulfation, releasing saccharide products with appreciable size (4–7 kDa) that can still associate with protein ligands and facilitate their biological potency. Mammalian cells express primarily a single dominant functional heparanase enzyme (heparanase-1).^16^,^17^,^37^,^38^ A second heparanase (heparanase-2) has been cloned and sequenced but has not been shown to have HS-degrading activity.^39^ For simplification, throughout this review we will refer to heparanase-1 as heparanase. Enzymatic degradation of HS leads to disassembly of the ECM and is therefore involved in fundamental biological phenomena associated with tissue remodeling and cell migration, including cancer angiogenesis and metastasis.^16^,^17^,^37^,^38^ The heparanase mRNA encodes a 61.2-kDa protein with 543 amino acids. This proenzyme is post-translationally cleaved into 8 and 50 kDa subunits that non-covalently associate to form the active heparanase (Figure 2).^17^,^38^,^40^ Heterodimer formation is essential for heparanase enzymatic activity.^40^,^41^ Site-directed mutagenesis revealed that, similar to other glycosyl hydrolases, heparanase has a common catalytic mechanism that involves two conserved acidic residues, a putative proton donor at Glu_225_, and a nucleophile at Glu_343_ (Figure 2).^42^ Cellular processing of the latent 65-kDa proheparanase into its active 8+50-kDa heterodimer is inhibited by a cell-permeable inhibitor of cathepsin L.^43^ Moreover, multiple site-directed mutagenesis and cathepsin L gene-silencing and knock-out experiments indicate that cathepsin L is the predominant enzyme responsible for processing and activation of proheparanase.^44^

## HEPARANASE IN TUMOR ANGIOGENESIS AND METASTASIS

**Figure 3:**
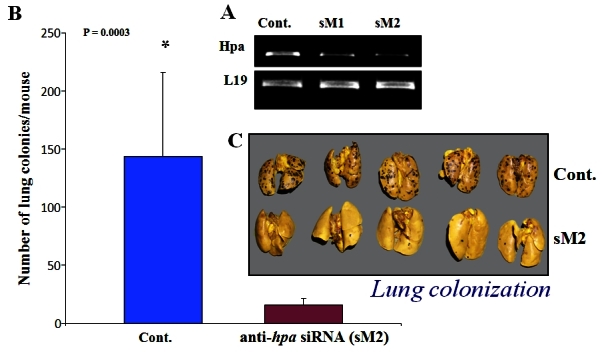
Lung colonization of B16 mouse melanoma cells is inhibited following silencing (sM2 antiheparanase siRNA) of the heparanase gene. Both gene expression (A: RT-PCR) and lung metastasis (B, C) are inhibited by 80%–90% upon silencing of the endogenous heparanase gene, indicating a causal involvement of heparanase in tumor cell metastasis.

Heparanase endoglycosidase activity was first demonstrated to be associated with the metastatic potential of tumor-derived cells such as B16 melanoma^45^ and T lymphoma.^46^ These early observations gained substantial support when specific molecular probes became available shortly after cloning of the heparanase gene. Both over-expression and silencing (Figure 3) of the heparanase gene clearly indicate that heparanase not only enhances cell dissemination but also promotes the establishment of a vascular network that accelerates primary tumor growth and provides a gateway for invading metastatic cells.^16^ While these studies provided a proof-of-concept for the prometastatic and proangiogenic capacity of heparanase, the clinical significance of the enzyme in tumor progression emerged from a systematic evaluation of heparanase expression in primary human tumors. Heparanase has been found to be up-regulated in essentially all human carcinomas and sarcomas examined.^16^ Notably, increased heparanase levels were most often associated with reduced patient survival *post* operation, increased tumor metastasis, and higher microvessel density.^16^,^47^

The cellular and molecular mechanisms underlying enhanced tumor growth by heparanase are only starting to be revealed. At the cellular level, both tumor cells and cells that comprise the tumor microenvironment (i.e. endothelial, fibroblasts, tumor-infiltrating immune cells) are likely to be affected by heparanase. Proangiogenic potency of heparanase was established clinically^16^,^48^ and in several *in-vitro* and *in-vivo* model systems, including wound-healing,^49^,^50^ tumor xenografts,^51^ Matrigel plug assay,^49^ and tube-like structure formation. Moreover, microvessel density was significantly reduced in tumor xenografts developed by T lymphoma cells transfected with antiheparanase ribozyme.^52^ The molecular mechanism by which heparanase facilitates angiogenic responses has traditionally been attributed primarily to the release of HS-bound growth factors such as VEGF-A and FGF-2,^18^,^53^ a direct consequence of heparanase enzymatic activity.

Heparanase was also noted to facilitate the formation of lymphatic vessels. In head and neck carcinoma, high levels of heparanase were associated with increased lymphatic vessel density (LVD), increased tumor cell invasion to lymphatic vessels, and increased expression of VEGF-C,^54^ a potent mediator of lymphatic vessel formation. Heparanase over-expression by melanoma, epidermoid, breast and prostate carcinoma cells induced a 3–5-fold elevation of VEGF-C expression *in vitro*, and facilitated lymph angiogenesis of tumor xenografts *in vivo*, whereas heparanase gene silencing was associated with decreased VEGF-C levels.^54^ Importantly, active heparanase does not completely digest the HS chains it attacks; rather, it cleaves the glycosidic bonds of HS chains at only a few sites, producing fragments that are 10–20 sugar residues long.^55^ There is evidence that the fragments of HS generated by heparanase are more biologically active than the native HS chain from which they are derived.^49^,^56^ Thus, heparanase acts as an “activator” of HSPGs and therefore is a pivotal player in creating a growth-permissive microenvironment for tumor growth. These and other results^57^,^58^ strongly suggest that heparanase and HSPGs act synergistically within the tumor microenvironment to enhance tumor growth, implying that inhibitors of heparanase will benefit cancer patients.

## HEPARANASE AND HEPARAN SULFATE IN INFLAMMATION

Up-regulation of heparanase was reported in different inflammatory conditions, often associated with degradation of HS and release of chemokines anchored within the ECM network and cell surfaces. Moreover, remodeling of the ECM facilitates transmigration of inflammatory cells towards the injury sites. Prior to cloning of the heparanase gene, heparanase activity originating in activated cells of the immune system (T lymphocytes, neutrophils) has been found to contribute to their ability to penetrate blood vessel and accumulate in target organs.^59^ More recently, it was demonstrated that up-regulation of heparanase, locally expressed (i.e. by vascular endothelium, skin keratinocytes) at the site of inflammation, is an essential step of delayed-type hypersensitivity (DTH).^60^ Degradation of HS in the subendothelial basement membrane resulted in vascular leakage, a hall-mark of DTH skin reactions.^60^ Up-regulation of heparanase has also been found in colonic epithelium of patients with inflammatory bowel disease (IBD) both at the acute and chronic phases of the disease,^61^ and in skin lesions of psoriasis patients (our unpublished results). Notably, heparanase staining was primarily detected in epithelial rather than immune cells, indicating that heparanase levels are elevated under chronic inflammatory conditions and autoimmunity. Heparanase activity was also found to be dramatically elevated in synovial fluid from rheumatoid arthritis (RA) patients,^62^ suggesting an important role for heparanase in promoting joint destruction and indicating heparanase as an attractive target for the treatment of RA.^62^

In line with findings observed with *Ndst1* mutant cells, it was demonstrated that a majority of intravascular neutrophils crawled toward and transmigrated closer to a chemokine-releasing gel that was placed beside the vessel.^63^ This directional crawling was absent in heparanase transgenic (*hpa-tg*) mice, which express shorter HS chains because of heparanase over-expression. This resulted in random crawling and decreased leukocyte recruitment in the *hpa-tg* versus wild-type mice and ultimately a severely reduced ability to clear a bacterial infection. It was concluded that a chemokine gradient is formed along intact HS on the endothelium and that this intravascular gradient effectively directs crawling leukocytes toward transmigration sites adjacent to the site of infection.

## NON-ENZYMATIC FUNCTIONS

Enzymatically inactive heparanase was noted to facilitate adhesion and migration of primary endothelial cells^64^ and to promote phosphorylation of signaling molecules such as Akt and Src,^64^,^65^ the latter found responsible for VEGF-A induction following exogenous addition of heparanase or its over-expression.^66^ The concept of enzymatic activity-independent function of heparanase gained substantial support by the identification of the heparanase C-terminus domain (C-domain) (Figure 2) as the molecular determinant behind its signaling capacity. The existence of a C-domain emerged from a prediction of the three-dimensional structure of a single-chain heparanase enzyme.^67^ In this protein variant, the linker segment was replaced by three glycine-serine repeats (GS3), resulting in a constitutively active enzyme.^41^ The structure obtained clearly illustrates a triosephosphate isomerase (TIM)-barrel fold, in agreement with previous predictions.^42^,^43^ Notably, the structure also delineates a C-terminus fold positioned next to the TIM-barrel fold (Figure 2).^67^ The predicted heparanase structure led to the hypothesis that the seemingly distinct protein domains observed in the three-dimensional model, namely the TIM-barrel and C-domain regions, mediate enzymatic and non-enzymatic functions of heparanase, respectively. Interestingly, cells transfected with the TIM-barrel construct (amino acids 36–417) failed to display heparanase enzymatic activity, suggesting that the C-domain is required for the establishment of an active heparanase enzyme, possibly by stabilizing the TIM-barrel fold.^67^ Deletion and site-directed mutagenesis further indicated that the C-domain plays a decisive role in heparanase enzymatic activity and secretion.^67^–^69^ Notably, Akt phosphorylation was stimulated by cells over-expressing the C-domain (amino acids 413–543), while the TIM-barrel protein variant yielded no Akt activation compared with control, mock transfected cells.^67^ These findings indicate that the non-enzymatic signaling function of heparanase leading to activation of Akt is mediated by the C-domain. Notably, the C-domain construct lacks the 8-kDa segment (Gln_36_-Ser_55_) which, according to the predicted model, contributes one beta strand to the C-domain structure (reviewed by Fux et al.^67^). Indeed, Akt phosphorylation was markedly enhanced and prolonged in cells transfected with a mini-gene comprising this segment linked to the C-domain sequence (8-C).^67^ The cellular consequences of C-domain over-expression were best revealed by monitoring tumor xenograft development. Remarkably, tumor xenografts produced by C-domain-transfected glioma cells grew faster and appeared indistinguishable from those produced by cells transfected with the full-length heparanase in terms of tumor size and angiogenesis, yielding tumors 6-fold bigger than control. In contrast, progression of tumors produced by TIM-barrel-transfected cells appeared comparable with control mock transfected cells.^67^ These results show that in some tumor systems (i.e. glioma) heparanase facilitates primary tumor progression regardless of its enzymatic activity, while in others (i.e. myeloma) heparanase enzymatic activity dominates (see below). Enzymatic activity-independent function of heparanase is further supported by the recent identification of T5, a functional human splice variant of heparanase.^70^

The emerging signaling capacity of heparanase should not come as a surprise. Enzymatic activity-independent function has been described for diverse classes of enzymes including, among others, caspases,^71^ cathepsins,^72^ plasminogen activator,^73^ matrix metalloproteinases (MMPs),^10^ and even telomerase.^74^ MMPs are a family of 23 zinc-dependent mammalian metalloenzymes which, after processing to their active form, are able to cleave all known ECM components. ECM degradation by MMPs has long been implicated in cellular invasion and metastasis, yet MMPs inhibitors failed as anticancer therapeutics.^75^ The reason behind this disappointing conclusion combines several considerations,^75^ among which is the increasing awareness of a non-proteolytic function of MMPs which is not affected by MMP inhibitors.^10^ It is now evident that MMP function is not restricted to cleavage of ECM constituents but rather MMPs are also engaged in multiple signaling pathways that affect the tumor cells and the tumor microenvironment. Non-proteolytic function of MMPs is thought to be executed primarily by their C-terminal, hemopexin-like domain. For example, the hemopexin domain of MMP-9 but not its proteolytic activity is necessary for enhanced epithelial cell migration, mediated by the PI3-kinase pathway.^76^ Likewise, the hemopexin domain of MMP-9 attenuated apoptosis of leukemia cells in a Src-dependent manner. Thus, apart from their well characterized enzymatic activity function in cancer metastasis and angiogenesis, the status of heparanase and MMP research parallels in terms of concept (enzymatic activity-independent function), methodology (i.e. transfection of catalytically inactive mutants), cellular consequences (i.e. increased cell adhesion and migration). For both MMPs and heparanase the underlying molecular mechanism (i.e. PI3-kinase and Src activation) is executed by the C-terminus domains (hemopexin and C-domain, respectively).^67^ This and other examples^71^,^72^ suggest that enzyme function exceeds beyond the enzymatic aspect, thus significantly expanding the scope of the functional proteome.

## HEPARANASE INHIBITION STRATEGIES

Attempts to inhibit heparanase enzymatic activity were initiated already in the early days of heparanase research, in parallel with the emerging clinical relevance of this activity. More recently, with the availability of recombinant heparanase and the establishment of high-throughput screening methods, a variety of inhibitory molecules have been developed, including neutralizing antibodies, peptides, small molecules, modified non-anticoagulant species of heparin, as well as several other polyanionic molecules, such as suramin and PI-88.^77^,^78^ Suramin, a sulfonated naphthylurea, has multiple antitumor effects (including an ability to block heparanase activity) but causes relatively severe side-effects in humans.^79^ PI-88 is a yeast-derived phosphosulfomannan that performed well in phase I and II clinical trials, exhibiting efficacy against several cancers.^80^ In addition to blocking heparanase activity, it also interferes with growth factor interactions, leading to inhibition of angiogenesis.^81^ However, because PI-88 is a complex mixture of oligosaccharides, characterization of its structure-activity relationships has been complicated, thereby necessitating attempts to generate analogs with desirable pharmacokinetic properties.^82^ A significant progress is represented by the PG500 series, a collection of new HS mimetics based on anomerically pure, fully sulfated, oligosaccharide glycosides modified by the addition of an aglycone at the reducing end of the molecule.^82^ The aglycones are primarily lipophilic groups chosen specifically to improve the biological activities, primarily the efficacy and pharmacokinetic properties. PG500 series compounds are believed to interfere with two important processes in tumor development, namely angiogenesis via inhibition of VEGF, FGF-1, and FGF-2, and metastasis via inhibition of heparanase activity. Compound PG545 was tested in a HT29 colon xenograft model and found to inhibit markedly tumor development comparable with the standard of care chemotherapeutic agent 5-fluorouracil (5-FU). The fact that administration of these agents to tumor-bearing animals led to significant tumor growth inhibition strongly supports further development of these HS mimetics for the treatment of cancer.

**Figure 4:**
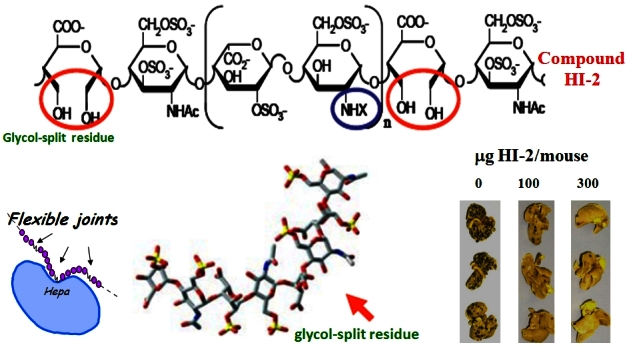
A chemically modified, non-anticoagulant heparin is a potent inhibitor of heparanase enzymatic activity and melanoma lung colonization. Structure (top) and favored 3D conformation (bottom) of heparanase inhibitor 2 (HI-2) = heparin that is glycol-split (denoted by red circle) and N-acetylated (denoted by the blue circle). The enhanced flexibility of glycol-split heparin facilitates tight binding to heparanase (Hepa, bottom, left), resulting in potent inhibition of the enzyme activity and melanoma lung colonization (bottom, right).

Heparin is a potent inhibitor of heparanase, but its use at high doses is impossible due to the potential for anticoagulant activity.^83^ Interestingly, low-molecular-weight heparin (LMWH), being more bioavailable and less anticoagulant than heparin, appears to prolong survival of patients with cancer. In several randomized controlled trials, four different types of LMWH increased the survival of patients with advanced cancer.^84^ Indeed, rather than just preventing fatal pulmonary emboli in cancer patients, it seems more likely that LMWH has direct effects on tumor growth and metastasis. This may be due, at least in part, to inhibition of heparanase enzyme activity by LMWH. On the basis of the structure-activity relationship emerging from our heparanase inhibition studies and in view of clinical data on the anticancerous and anti-inflammatory effect of heparin,^84^ we initiated a systematic study aimed at obtaining heparanase-inhibiting species of heparin devoid of anticoagulant and proangiogenic activities. In performing these experiments, we have noted a pronounced gain of heparanase-inhibiting activity following glycol-splitting of both the N-sulfated and N-acetylated forms of heparin.^48^,^85^ Glycol-split residues act as carboxylated, flexible joints along the sulfated polysaccharide chains, thereby strengthening their binding to heparanase (Figure 4). This facilitates the best fit between the glycol-split molecule and the two basic heparin/HS-binding sites of heparanase. Heparin that is 100% N-acetylated and 25% glycol-split (which we have named heparanase inhibitor-2 (HI-2)) (Figure 4) was found to be an especially strong and specific inhibitor of heparanase, yielding 100% inhibition of its enzymatic activity at 10 nanomolar concentrations *in vitro*. Since glycol splitting also involves inactivation of the active site for antithrombin, compound HI-2 exhibits a very low or no anticoagulant activity. We have demonstrated the effectiveness of glycol-split heparinoids, including compound HI-2 (=^100^NA,R.OH), in suppressing the biological activity of heparanase, applying *in-vivo* models of inflammation,^60^ melanoma lung colonization (Figure 4),^86^ and myeloma tumor growth.^58^,^83^

Random, high-throughput screening of chemical libraries and microbial metabolites and rational design of compounds that block the heparanase active site or ligand-binding domain are among the approaches applied to develop effective heparanase inhibitors.^77^,^78^ Natural endogenous heparanase inhibitors may also be identified. Further defining the heparanase substrate specificity, catalytic and non-catalytic activities, as well as the enzyme crystal structure is needed for pursuing a more “rational” approach to develop effective and highly specific heparanase inhibiting molecules.

## MOVING ANTIHEPARANASE THERAPY CLOSER TO REALITY

Multiple myeloma is the second most prevalent hematologic malignancy. This B lymphoid malignancy is characterized by tumor cell infiltration of the bone-marrow, resulting in severe bone pain and osteolytic bone disease. Although progress in the treatment of myeloma patients has been made over the last decade, the overall survival of patients is still poor. In myeloma patients, heparanase enzymatic activity was elevated in the bone-marrow plasma of 86% of patients examined,^87^ and gene array analysis showed elevated heparanase expression in 92% of myeloma patients.^57^ Heparanase up-regulation in myeloma patients was associated with elevated microvessel density and syndecan-1 expression.^87^ While heparanase is proangiogenic in myeloma, which is a common feature shared with solid tumors, heparanase regulation of syndecan-1 shedding has emerged as highly relevant to multiple myeloma progression.

Syndecan-1 is particularly abundant in myeloma and is the dominant and often the only HSPG present on the surface of myeloma cells.^88^ Cell surface syndecan-1 promotes adhesion of myeloma cells and inhibits cell invasion *in vitro*.^89^ In contrast, high levels of shed syndecan-1 are found in the serum of some myeloma patients and are associated with poor prognosis.^90^ Notably, heparanase up-regulates both the expression and shedding of syndecan-1 from the surface of myeloma cells.^57^,^91^ In agreement with this notion, heparanase gene silencing was associated with decreased levels of shed syndecan-1.^57^ Importantly, both syndecan-1 up-regulation and shedding require heparanase enzymatic activity,^91^ suggesting that cleavage of HS by heparanase renders syndecan-1 more susceptible to proteases mediating the shedding of syndecan-1. However, it appears that heparanase may play an even more direct role in regulating shedding of syndecan-1, by facilitating the expression of proteases engaged in syndecan shedding.

It was recently demonstrated that enhanced expression of heparanase leads to increased levels of MMP-9 (a syndecan-1 sheddase), while heparanase gene silencing resulted in reduced MMP-9 activity.^92^ Moreover, not only MMP-9 but also urokinase-type plasminogen activator (uPA) and its receptor (uPAR), molecular determinants responsible for MMP-9 activation, are up-regulated by heparanase. These findings provided the first evidence for co-operation between heparanase and MMPs in regulating HSPGs on the cell surface and likely in the ECM and are supported by the recent generation and characterization of heparanase knock-out (KO) mice. Despite the complete lack of heparanase gene expression and enzymatic activity, heparanase-KO mice develop normally, are fertile, and exhibit no apparent anatomical or functional abnormalities.^93^ Notably, heparanase deficiency was accompanied by a marked elevation of matrix metalloproteinase (MMP) family members such as MMP-2, MMP-9, and MMP-14, in an organ-dependent manner, suggesting that MMPs provide tissue-specific compensation for heparanase deficiency. Collectively, these results suggest that heparanase is intimately engaged in the regulation of gene transcription and acts as a master regulator of protease expression, mediating gene induction or repression depending on the biological setting.

Results from studies using several *in-vivo* model systems support the notion that enzymatic activities responsible for syndecan-1 modification are valid targets for myeloma therapy. For example, enhanced expression of either HSulf-1 or HSulf-2 attenuated myeloma tumor growth.^94^ An even more dramatic inhibition of tumor growth was noted following administration of bacterial heparinase III (heparitinase) to SCID mice inoculated with myeloma cells isolated from the bone-marrow of myeloma patients.^58^ Unlike the bacterial enzyme, heparanase cleaves HS more selectively and generates fragments that are 4–7 kDa in size, yielding strictly distinct outcomes in the context of tumor progression. While administration of heparinase III is associated with reduced tumor growth, heparanase activity is elevated in many hematological and solid tumors, correlating with poor prognosis and shorter postoperative survival rate. Thus, inhibition of heparanase enzymatic activity is expected to suppress tumor progression. To examine this in myeloma, a chemically modified heparin, which is 100% N-acetylated and 25% glycol-split, was tested. This flexible molecule is a potent inhibitor of heparanase enzymatic activity, lacks anticoagulant activity typical of heparin, and does not displace ECM-bound FGF-2 or potentiate its mitogenic activity.^48^,^85^ The modified heparin profoundly inhibits the progression of tumor xenografts produced by myeloma^58^,^83^ and Ewing’s sarcoma^95^ cells. These studies support the notion that heparanase enzymatic activity not only facilitates tumor metastasis but also promotes the progression of primary tumors.

## CONCLUSIONS AND PERSPECTIVE

Although much has been learnt in the last decade, the repertoire of heparanase functions in health and disease is only starting to emerge. Clearly, from activity implicated mainly in cell invasion associated with tumor metastasis, heparanase has turned into a multi-faceted protein that appears to participate in essentially all major aspects of tumor progression. Heparanase expression is elevated already at the early stages of human neoplasia. In the colon, heparanase gene and protein are expressed already at the stage of adenoma,^96^ and during esophageal carcinogenesis heparanase expression is induced in Barrett’s epithelium, an early event that predisposes patients to formation of dysplasia which may progress to adenocarcinoma.^97^ Heparanase expression at the early stages of tumor initiation and progression, and by the majority of tumor cells, can be utilized to turn the immune system against the very same cells. Accumulating evidence suggests that peptides derived from human heparanase can elicit a potent antitumor immune response, leading to lysis of heparanase-positive human gastric, colon, and breast carcinoma cells, as well as hepatoma and sarcoma cells.^98^,^99^ In contrast, no killing effect was noted towards autologous lymphocytes.^98^,^99^ Notably, the development of tumor xenografts produced by B16 melanoma cells was markedly restrained in mice immunized with peptides derived from mouse heparanase (i.e. aa 398–405; 519–526) compared to a control peptide in both immunoprotection and immunotherapy approaches.^99^ T-regulatory cells are frequently present in colorectal cancer patients; interestingly, T-regulatory cells against heparanase could not be found.^100^ Antiheparanase immunotherapy is thus expected to be prolonged and more efficient due to the absence of T suppressor cells. A related treatment approach is being tested in advanced metastasized breast cancer patients.^101^ While this immunotherapeutic concept, together with available heparanase inhibitors, is hoped to advance cancer treatment, the identification of single nucleotide polymorphisms (SNPs) associated with heparanase expression and increased risk for graft versus host disease following allogeneic stem cell transplantation^102^ offers a genetic concept which can potentially be translated into patient diagnosis. Studies in these directions, identification of heparanase receptor(s) mediating its signaling function, and elucidation of heparanase route and function in the cell nucleus, will advance the field of heparanase research and reveal its significance in health and disease.

While most attention was paid in recent years to heparanase function in tumor biology, emerging evidence indicates that heparanase is also engaged in several other pathological disorders. A most interesting example is the apparent role of heparanase in glomerular diseases.^103^ HSPGs are important constituents of the glomerular basement membrane (GBM) and its permselective properties.^11^ Loss of HSPGs was observed in several experimental and human glomerulopathies, including diabetic nephropathy, minimal change disease, and membranous glomerulophathy. In addition, expression of heparanase was up-regulated in the course of these diseases,^104^ likely destructing the permselective properties of HS. Notably, PI-88 (a heparanase inhibitor) was effective as an antiproteinuric drug in an experimental model.^105^ Heparanase is also causally associated with inflammatory conditions such as inflammatory bowel disease^61^ and rheumatoid arthritis,^62^ among other inflammatory conditions (Lerner et al., our unpublished results). Novel heparanase inhibitors such as glycol-split heparin or more advanced oligosaccharide-based compounds^48^ are hoped to enter the clinic and provide relief in diabetic, colitis, and cancer patients’ condition. Resolving the heparanase crystal structure will accelerate the development of effective inhibitory molecules and neutralizing antibodies, paving the way for advanced clinical trials in patients with cancer and other diseases involving heparanase.

## Abbreviations:

BM: basement membrane;
ECM: extracellular matrix;
GAG: glycosaminoglycans;
HS: heparan sulfate;
HSPGs: heparan sulfate proteoglycans;
LMWH: low-molecular-weight heparin;
MMP: matrix metalloproteinase;
TIM: triosephosphate isomerase;
VEGF: vascular endothelial growth factor.
